# High frame rate (∼3 Hz) circular photoacoustic tomography using single-element ultrasound transducer aided with deep learning

**DOI:** 10.1117/1.JBO.27.6.066005

**Published:** 2022-06-20

**Authors:** Praveenbalaji Rajendran, Manojit Pramanik

**Affiliations:** Nanyang Technological University, School of Chemical and Biomedical Engineering, Singapore

**Keywords:** photoacoustic tomography, high framerate imaging, deep learning, circular photoacoustic tomography

## Abstract

**Significance:**

In circular scanning photoacoustic tomography (PAT), it takes several minutes to generate an image of acceptable quality, especially with a single-element ultrasound transducer (UST). The imaging speed can be enhanced by faster scanning (with high repetition rate light sources) and using multiple-USTs. However, artifacts arising from the sparse signal acquisition and low signal-to-noise ratio at higher scanning speeds limit the imaging speed. Thus, there is a need to improve the imaging speed of the PAT systems without hampering the quality of the PAT image.

**Aim:**

To improve the frame rate (or imaging speed) of the PAT system by using deep learning (DL).

**Approach:**

For improving the frame rate (or imaging speed) of the PAT system, we propose a novel U-Net-based DL framework to reconstruct PAT images from fast scanning data.

**Results:**

The efficiency of the network was evaluated on both single- and multiple-UST-based PAT systems. Both phantom and *in vivo* imaging demonstrate that the network can improve the imaging frame rate by approximately sixfold in single-UST-based PAT systems and by approximately twofold in multi-UST-based PAT systems.

**Conclusions:**

We proposed an innovative method to improve the frame rate (or imaging speed) by using DL and with this method, the fastest frame rate of ∼3  Hz imaging is achieved without hampering the quality of the reconstructed image.

## 1 Introduction

Photoacoustic imaging (PAI) is a noninvasive hybrid imaging modality that combines the virtues of both optical and ultrasound imaging.^1^^–^^3^ Over the last decade, the potential of PAI has been exemplified through numerous clinical and preclinical studies.^4^^–^^11^ PAI relies on the photoacoustic (PA) effect for the generation of images. The PA effect is commonly induced by the irradiation of the target chromophores by nanosecond laser pulses. The absorption of incident light energy by the chromophores results in a local temperature rise, which leads to the generation and propagation of ultrasound waves (due to thermoelastic expansion and contraction), known as PA waves. These PA waves are then acquired around the boundary of the target by employing ultrasound detectors. In photoacoustic tomography (PAT)/photoacoustic computed tomography (PACT), typically, a single-element ultrasound transducer (UST) or transducer arrays are used as detectors. The acquired PA signals (also known as A-lines) are used to reconstruct the cross-sectional PAT images with different types of reconstruction algorithms.^12^^–^^14^

Conventionally, in PAT systems based on circular scanning geometry, a single UST is rotated 360 deg around the target to collect the A-lines.^15^ It takes several minutes to acquire the necessary number of A-lines for generating a PAT image of acceptable quality. Furthermore, the quality of PAT images improves with the increase in the number of A-lines acquired. However, with low pulse repetition rate (PRR) excitation sources (commonly used nanoseconds high energy pulsed lasers have PRR of 10 to 100), collecting a high enough number of A-lines for high-quality PAT image protracts to several minutes. Improving imaging speed for circular scan PAT system is important, as fast dynamic imaging is possible only with a fast-imaging system.^16^ Using an array UST is one way of improving the imaging speed.^17^^–^^19^ As with array detectors, there is no need for scanning and even with a single laser pulse, the entire cross-sectional imaging can be done.^20^ However, array-based ultrasound detectors often required custom-made array transducers, parallel multichannel data acquisition electronics making those systems bulky, very expensive, and cumbersome to use. Hence, building a single-element UST-based fast circular scanning PAT system is very important.

To improve the scanning speed several steps have been taken in the last few years. First, instead of stop-and-go scanning, continuous scan improves the scanning speed significantly.^21^ Combining continuous scan with high PRR lasers/light sources improve the imaging speed even further. Over the recent years, a new type of excitation source called pulsed laser diodes (PLD) is garnering a lot of popularity in PAT due to its high PRR, compact size, and low cost compact size in comparison with the conventional low PRR Nd:YAG lasers.^22^ A high-speed PLD-based desktop PAT imaging system capable of generating an image at 3 s has already been demonstrated,^22^ and its imaging speed has been further improved to 0.5 s (PLD-PAT-G2) by employing multiple USTs.^23^ Although, the techniques such as the use of multiple USTs, continuous scan, and high PRR laser enhances the scanning speed of PAT systems, improving the imaging speed beyond 0.5 s [or 2 frames per second (fps)] remains a challenge due to the emergence of blurring and streaking artifacts arising from the sparse signal (A-line) acquisition and low signal-to-noise (SNR) at higher imaging speeds. To fight against sparse sampling, an analytical anti-aliasing method has been proposed earlier.^24^ However, it is applicable for array transducer based PAT systems and may not be directly implemented and applied on PAT systems based on single-element UST. Furthermore, interpolation-based techniques have also been proposed to tackle sparse sampling.^25^ However, it is a time-consuming iterative process. Thus, there is a need for a technique to increase the imaging speed even further without compromising the image quality.

Deep learning (DL) is a class of machine learning where a wide range of neural networks are employed to enhance the quality of images.^26^^,^^27^ Especially, convolutional neural networks (CNN) are widely preferred due to their ability to solve complex image-related tasks. CNN-based DL networks have also been employed in PAI to overcome various limitations and challenges encountered in traditional image reconstruction algorithms.^28^^–^^33^ In general, the CNN-based DL approaches employed in PAT can be broadly classified into four categories: pre-processing, post-processing, direct-processing, and hybrid-processing.^34^ In the pre-processing approach, the acquired PA data are enhanced by feeding it into the CNN before image reconstruction;^35^^–^^37^ in the post-processing approach, the resultant image from the conventional reconstruction is fed into the CNN to improve the image quality;^38^^–^^40^ in the direct-processing approach, the CNN is utilized to directly map the initial pressure maps from the raw PA data;^41^^,^^42^ and in the hybrid-processing approach, the PAT image is reconstructed feeding both conventionally reconstructed image and raw PA data into the CNN.^43^^,^^44^ Among these approaches, the post-processing-based DL approach has been mostly preferred in PAT due to its superiority over the other approaches^45^ and is optimal for applications such as artifact removal and contrast enhancement.

In this work, a post-processing-based DL approach is proposed to improve the frame rate of PAT systems. A unique CNN-based DL architecture termed dense hybrid dense UNet (HD-UNet) has been applied to improve the frame rate by reconstructing high-quality PAT images from the data acquired at higher scanning speeds. The network was optimized using the simulated data and its performance was evaluated on both single- and multi-UST-PAT systems using the phantom and *in vivo* images. k-Wave MATLAB toolbox^46^ was used for generating the simulated dataset using numerical phantoms for the training purpose. In comparison with the highest imaging speeds achieved with 1-UST-PAT (30 s imaging speed)^22^ and multi-UST-PLD-PAT system, (0.5 s imaging speed),^23^ the DL approach enhances the imaging speed by approximately sixfold (5 s imaging speed) in 1-UST-PAT systems and approximately twofold (0.3 s imaging speed) in the multi-UST-PLD-PAT system. Here, we report the single-element UST-based PAT imaging capable of acquiring an image in 0.3 s (∼3  fps). Furthermore, a significant improvement in the image quality was also achieved along with the enhancement in imaging speed.

## 2 Methods

### 2.1 Proposed HD-UNet Architecture

Since its advent, U-Net-based CNN has been widely used in complex imaging-related tasks and it comprises contraction and expansion layers with skip connections resembling a symmetrical U-shape.^47^ However, for improved accuracy and performance in U-Net, extensional techniques are needed.^48^ A modified version of U-Net, called fully dense U-Net (FD-U-Net), was first proposed for artifact removal and was then attuned for various PAI applications.^49^^,^^50^ The FD-U-Net incorporates dense blocks in both the contracting and expansive layers to enable the learning of additional feature maps from the knowledge gained by previous layers through concatenation. Furthermore, the dense blocks increase the network’s depth without incrementing the number of layers. An enhanced version of FD-U-Net termed dense dilated U-Net (DD-U-Net) was then proposed for correcting the artifacts in three-dimensional (3D) PAT systems.^51^ The dense dilated blocks employed in the DD-U-Net uses atrous convolutions along with standard convolutions in the dense blocks to increase the receptive field to extract additional information. Furthermore, the incorporation of atrous convolutions in the dense blocks allows the CNN to learn multiscale features in an exponential means.^52^ However, a significant limitation with the DD-U-Net is the memory constraint due to a large number of training parameters and gridding artifacts if dilated convolutions of large receptive fields are employed.^51^ Thus, for improving the frame rate of the PAT systems, we developed a DL architecture HD-UNet by leveraging the benefits of both dilated convolution and standard convolution.

**Fig. 1:**
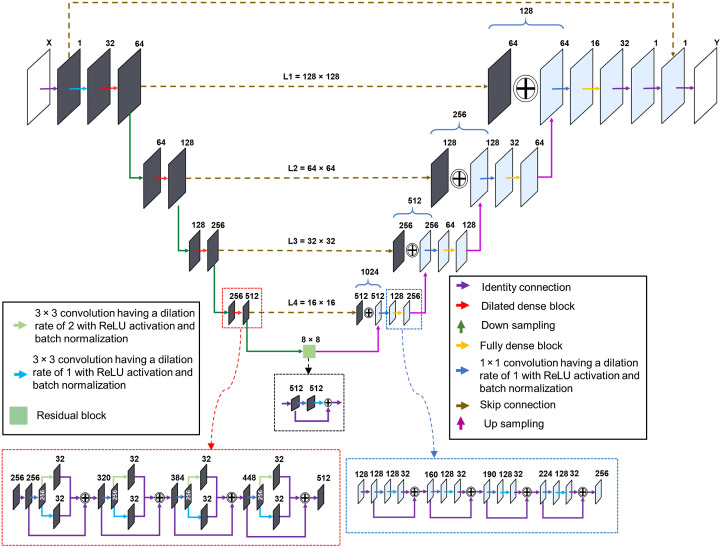
Schematic of the proposed HD-UNet architecture incorporating dense blocks with dilated convolution in contracting path and dense blocks with standard convolutions in expanding path. L1, L2, L3, and L4 refers to the different layer levels. X and Y are the input and output image of size 128×128  pixels.

The proposed network incorporates dilated dense blocks in the encoding path followed by the standard dense blocks in the decoding path along with a residual block as the bridge. The schematic of the HD-UNet architecture is shown in Fig. 1. Depending on the layer level (l), the dense block employed in the encoding path intends to learn $f_{l}$ feature maps from the input feature map $f_{i}$ by iteratively learning $k_{l}$ features maps at each step. The standard dense blocks employed in the expanding path learns $f_{l}=2^{l-1}\times f_{i}$ at the growth factor of $k_{l}=2^{l-1}\times 8$ using 3×3 convolutions of dilation rate 1. On the encoding path, the dilated convolutions implemented also learn $k_{l}$ features and it can be represented as $k_{l}={\left[\frac{k_{l}}{2}\right]}_{s}+{\left[\frac{k_{l}}{2}\right]}_{d}$, where ${\left[\frac{k_{l}}{2}\right]}_{d}$ refers to features from convolutions with dilation rates 1 (standard convolution) and ${\frac{k_{l}}{2}}_{s}$ are from convolutions with dilation rate 2 (dilated convolution). The dilation rate is limited to 2 for reducing the gridding artifacts. The down sampling operation in the encoding path is carried out by a 1×1 convolution block followed by a 3×3 convolution block of stride 2 and the up-sampling operation in the decoding path is performed by a transposed 3×3 convolution block of stride 2. Skip connections were also implemented at each level to prevent the loss of any spatial information. Two 3×3 convolution blocks were employed at the end of decoding to generate the resultant image. Each convolution block used in the model consists of batch normalization preceded by convolution and rectified linear unit (RELU) activation [RELU(x)=max{x,0}, where x is the input to the neuron]. The proposed HD-UNet accepts an input image X of size 128×128  pixels and generates an output image Y of size 128×128  pixels.

### 2.2 Network Optimization and Implementation

The HD-UNet was implemented in Python 3.9 using the Tensorflow (V2.7) DL library.^53^ The optimization of the network was performed on an Nvidia Tesla V100-32 GB GPU using the nodes of the Gekko cluster, High-Performance Computing Centre, Nanyang Technological University, Singapore. Adam optimizer with a call-back monitor reducing the learning rate by a factor of 0.5 on instances of no improvement in the monitored metrics was used. The initial learning rate was set to be 0.001. The loss function employed in the model is a composite of two-loss functions with weights $k_{1}$ and $k_{2}$ and the equation is expressed as

$$L=k_{1}L_{\mathrm{MAE}}+k_{2}L_{\mathrm{FMAE}},$$

where $L_{\mathrm{MAE}}$ is the mean absolute error (MAE) and it aims to reduce the pixel-wise difference between the ground truth $Y_{g}$ and predicted image $Y_{p}$, the related equation is

$$L_{\mathrm{MAE}}(Y_{g},Y_{p})=\frac{1}{N}\overset{N}{\underset{i=1}{\sum }}|Y_{g}-Y_{p}|.$$

The $L_{\mathrm{FMAE}}$ is the Fourier mean absolute error loss (FMAE) and is applied to enforce the pixel-wise similarity between the ground truth $Y_{g}$ and predicted image $Y_{p}$, which is given by

$$L_{\mathrm{FMAE}}(Y_{g},Y_{p})=\frac{1}{N}\overset{N}{\underset{i=1}{\sum }}|F(Y_{g})-F(Y_{p})|.$$

The weights $k_{1}$ and $k_{2}$ used for optimizing the network are 1 and 0.001, respectively. The weights were chosen in such a way that the pixel-wise MAE serves as the primary loss. As the FMAE can contribute to instability in training, a smaller weighing factor was chosen. In total, the model was trained for 100 epochs with a batch size of two, and its performance was evaluated after the training.

### 2.3 Simulated Photoacoustic Datasets for Training

**Fig. 2:**
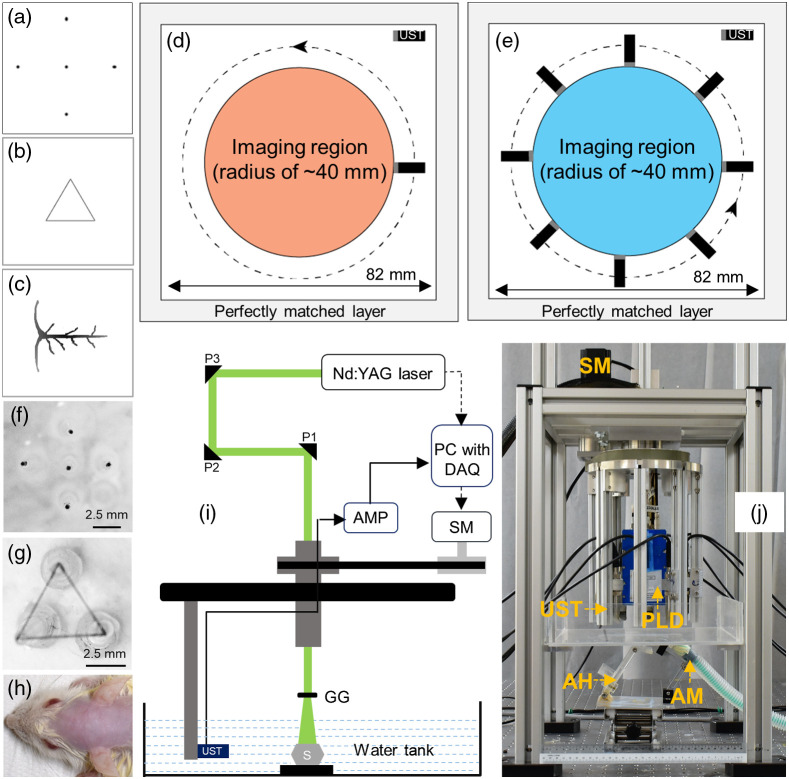
(a) Five-point sources numerical phantom. (b) Triangular numerical phantom. (c) Numerical blood vessel phantom. (d) Schematic of the k-wave simulation geometry of 1-UST-PAT system, (e) simulation geometry of the 8-UST-PAT system. (f) Five-point source phantom made up of pencil leads. (g) Triangular phantom made up of horsehair. (h) Photograph of the rat brain area used for *in vivo* imaging. (i) Schematic of the 1-UST-PAT system employed for phantom imaging. (j) Photograph of the 8-UST-PLD-PAT system used for *in vivo* brain imaging. AMP, amplifier; SM, stepper motor; DAQ, data acquisition card; PC, personal computer; UST, ultrasound transducer; AM, anesthesia machine; AH, animal holder; GG, ground glass; P1, P2, and P3 are uncoated prisms; S is the imaging sample.

DL is a data-based optimization approach, and its performance relies on the quality of the training data. In general, the training dataset used for optimizing the model comprises an input image and ground truth image. Although it is viable to generate a large amount of input data experimentally, the ground truth experimental data are sometimes difficult to obtain. Thus, for optimizing the HD-UNet, we used the simulated dataset with k-Wave MATLAB toolbox^46^ for training the model. For generating the simulated data to improve the frame rate in the 1-UST-PAT system, three numerical phantoms such as: five-point targets (in which the position, orientation, and source strength of the point sources were varied randomly), triangles (in which the position, orientation, and size of the triangles were varied randomly), and vessel shapes-mimicking the cerebral venous sinuses of the rodent brain (in which the orientation, magnitude, and position were varied randomly) were used [Figs. 2(a)–2(c)]. A computational grid of 82×82  mm (0.2  mm/pixel) and a perfectly matched bounding layer were used for the simulation [Fig. 2(d)]. The imaging region was constrained to 40 mm. The SNR was maintained at 40 dB and 40 ns step size with 1500-time steps was used. The medium chosen was acoustically homogeneous and the speed of sound used is 1500  m/s. For generating the input PA data, the number of detector positions (sensor points) was randomly varied between 10 and 50 at steps of 10 (∼1 to 5 s scanning time), a large aperture unfocused UST (13 mm active area) of central frequency 2.25 MHz with 70% nominal bandwidth was used as the detector. For the ground truth data generation, 4800 detector positions with an ideal point detector of central frequency 2.25 MHz and 70% nominal bandwidth were considered.

For improving the frame rate of the 8-UST-PAT system, vessel shapes resembling the rodent cerebral sinuses were used. A computational grid consisting of 82×82  mm (0.1  mm/pixel) and a perfectly matched bounding layer were considered. The schematic of the computational grid is shown in Fig. 2(e). The SNR was maintained at (10 to 20 dB). 1500-time steps with a step size of 40 ns were used for recording the A-lines. The medium used is acoustically homogeneous and the speed of sound was maintained at 1500  m/s. For the input data generation, eight large aperture unfocused UST (5 MHz central frequency with 70% nominal bandwidth) with 240 detector positions (30 detector locations per UST) were used. For the generation of ground truth data, an ideal point detector (5 MHz central frequency and 70% nominal bandwidth) with 1600 detector positions was considered. In both cases, conventional delay-and-sum beamformer was employed to reconstruct the PA data into cross-sectional PAT images of size 128×128  pixel. The reconstructed PAT images were then normalized by rescaling it in the new range of 0 to 1 without the loss of bipolar information using the equation,

$$A_{\text{out}}=\frac{\left[A_{ij}-A_{\min}\right]}{\left[A_{\max}-A_{\min}\right]},$$

where $A_{\min}$ is the minimum value of the array, $A_{\max}$ is the maximum value of the array, $A_{ij}$ is the value of the array with respect to the coordinates, and $A_{\text{out}}$ is the normalized array. For the 1-UST-PAT system, 1500 PAT images were generated, and for the 8-UST-PAT system, 500 PAT images were generated. They were randomly divided into training, validation, and testing set in the ratio of 90:5:5. The training dataset was used for optimizing the network, the validation dataset was used for tuning the hyperparameters, and the testing dataset is used for the performance evaluation of the network. Depending on the intended application (configuration of PAT system), the HD-UNet was optimized and evaluated using the respective dataset.

### 2.4 Experimental Phantom Data

Experimental phantom imaging was performed for evaluating the performance of the optimized HD-UNet. For obtaining the experimental phantom data, two types of phantoms namely, five-point targets (made up of pencil leads) [Fig. 2(f)] and triangular phantom (made up of horsehair) [Fig. 2(g)] were used. 1-UST-PAT system was employed for phantom PAT imaging.^54^ The schematic of the 1-UST-PAT system is shown in Fig. 2(i). A Q-switched Nd:YAG laser delivering 532 nm laser pulses with 10 pulses per second at a pulse width of 5 ns was employed as the excitation source. The emergent laser beam was homogenized using an optical diffuser and the laser energy density was maintained at $∼6\;\mathrm{mJ}/{\mathrm{cm}}^{2}$. An unfocused UST of 2.25 MHz (Olympus-NDT, V306-SU) central frequency (70% nominal bandwidth) was used to acquire the PA signals. An ultrasound pulse-receiver (Olympus-NDT, 5072PR) with a gain of 48 dB is used to amplify the PA signals. The amplified PA signals were then stored inside the desktop computer using a data acquisition card (DAQ) [GaGe, compuscope 4227]. Conventional delay-and-sum beamformer was used to reconstruct the cross-sectional PAT images from the PA data.

### 2.5 *In Vivo* Experimental Data

The performance of the proposed HD-UNet was also evaluated on the *in vivo* PAT imaging. Sprague Dawley rats (∼95  gm) obtained from InVivos Pte. Ltd., Singapore were utilized for imaging [Fig. 2(h)]. The rats were anesthetized by the intraperitoneal administration of ketamine (100  mg/mL) and xylazine (20  mg/mL) mixture. The hair on the rat head was then removed using depilatory cream, and the ocular gel was applied before imaging. A layer of ultrasound was applied to the scalp and a constant supply of anesthesia (1.0  L/min oxygen and 0.75% isoflurane) was maintained during imaging. All the animal experiments were performed as per the guidelines of the Institutional Animal Care and Use Committee, Nanyang Technological University, Singapore (Protocol No.: A0331). The *in vivo* PAT imaging was performed using the 8-UST-PLD-PAT system.^23^ The image of the 8-UST-PLD-PAT system is shown in Fig. 2(j). A PLD capable of delivering ∼816  nm wavelength at 2000 pulses per second with a pulse width of ∼107  ns and 3.4 mJ per pulse energy was used as the excitation source. An optical diffuser was employed to homogenize the emergent rectangular beam from the PLD, and the laser energy density was maintained at $∼0.17\;\mathrm{mJ}/{\mathrm{cm}}^{2}$, below the safety limits of American National Standards Institute (ANSI).^55^ Eight unfocused USTs (5 MHz central frequency with 70% nominal bandwidth) fitted with 45 deg acoustic reflectors (Olympus-NDT, F102) were employed to acquire the PA data. The PA signals were then amplified using a 48 dB low signal noise amplifier (Mini-circuits, ZFL-500LNBNC, two of them in series each with 24 dB gain) and stored inside a computer (IntelXeon, 3.7 GHz 64-bit processor, 16 GB RAM) using a DAQ card (Spectrum, M2i.4932-Exp). Conventional delay-and-sum beamformer was used to reconstruct the cross-sectional PAT brain images.

For *in vivo* imaging, the maximum permissible exposure (MPE) is limited by the ANSI laser safety standards.^55^ For the wavelength in the range of 700 to 1050 nm, the maximum per pulse energy density on the skin surface should not exceed $20\times 10^{2(\lambda -700)/1000}\;\mathrm{mJ}/{\mathrm{cm}}^{2}$. For 816 nm wavelength, the MPE is $∼34.12\;\mathrm{mJ}/{\mathrm{cm}}^{2}$. For the illumination period of 1.5 s (t=1.5  s), the MPE safety limit is $1.1\times 10^{2(\lambda -700)/1000}\times t^{0.25}\;J/{\mathrm{cm}}^{2}$ ($=2.07\;J/{\mathrm{cm}}^{2}$). Thus, for the scan time of 1.5 s, the MPE per pulse is $0.69\;\mathrm{mJ}/{\mathrm{cm}}^{2}$. Similarly, for a scan time of 0.3 s, the MPE safety limit is $1.39\;J/{\mathrm{cm}}^{2}$, and per pulse, it is $2.31\;\mathrm{mJ}/{\mathrm{cm}}^{2}$. As the per pulse energy was maintained at $∼0.17\;\mathrm{mJ}/{\mathrm{cm}}^{2}$ during the *in vivo* imaging (the PLD used in our study produces a max energy of ∼3.4  mJ per pulse, and it was illuminating $∼20\;{\mathrm{cm}}^{2}$ area), the per pulse energy does not exceed the ANSI safety limit for the scan time of 0.3 and 1.5 s.

## 3 Results

### 3.1 Performance Comparison

After the optimization, k-fold cross-validation (k=10) was implemented to evaluate the performance of the proposed HD-UNet, and it was compared with the performances of other DL architectures such as the FD-UNet, 2D-DD-UNet (an adapted version of 3D-DD-UNet^51^), and U-Net, using a variety of loss metrics such as Pearson correlation coefficient (PCC), structural similarity index measure (SSIM), peak signal-to-noise ratio (PSNR), and MAE. For the performance evaluation, the original dataset was randomly split 10 times in to into training, validation, and testing. For each dataset, the FD-Unet, 2D-DD-UNet, and U-Net were optimized for 100 epochs and its performance was evaluated on the testing dataset. On evaluation, the HD-UNet exhibited superior performance over the FD-UNet, 2D-DD-UNet, and U-Net over all the metrics (Table 1) and it signifies the generalizability of the developed HD-UNet.^56^

**Table 1:** k-Fold cross-validation (k=10) to compare the performance of the HD-UNet (mean ± standard deviation). The best values are shown in bold.

	k-Fold cross validation (k=10)				
	Network	PCC	PSNR	SSIM	MAE
1-UST-PAT	HD-UNet	**∼0.92 ± 0.14**	**∼35.02 ± 6.00**	**∼0.99 ± 0.03**	**∼0.017 ± 0.015**
	FD-UNet	∼0.80 ± 0.21	∼33.22 ± 5.22	∼0.97 ± 0.04	∼0.020 ± 0.016
	2D-DD-UNet	∼0.64 ± 0.25	∼26.76 ± 5.99	∼0.78± 0.13	∼0.050 ± 0.053
	UNet	∼0.58 ± 0.31	∼28.67 ± 6.32	∼0.92 ± 0.10	∼0.025 ± 0.101
8-UST-PAT	HD-UNet	**∼0.94± 0.02**	**∼32.90 ± 2.54**	**∼0.98 ± 0.01**	**∼0.017 ± 0.098**
	FD-UNet	∼0.92 ± 0.03	∼32.2 ± 2.33	∼0.96 ± 0.04	∼0.014 ± 0.096
	2D-DD-UNet	∼0.52 ± 0.11	∼20.77 ± 3.91	∼0.61± 0.07	∼0.048 ± 0.024
	UNet	∼0.73 ± 0.12	∼29.38 ± 5.06	∼0.94 ± 0.08	∼0.067 ± 0.153

### 3.2 Performance of HD-UNet on Simulated Phantoms

**Fig. 3:**
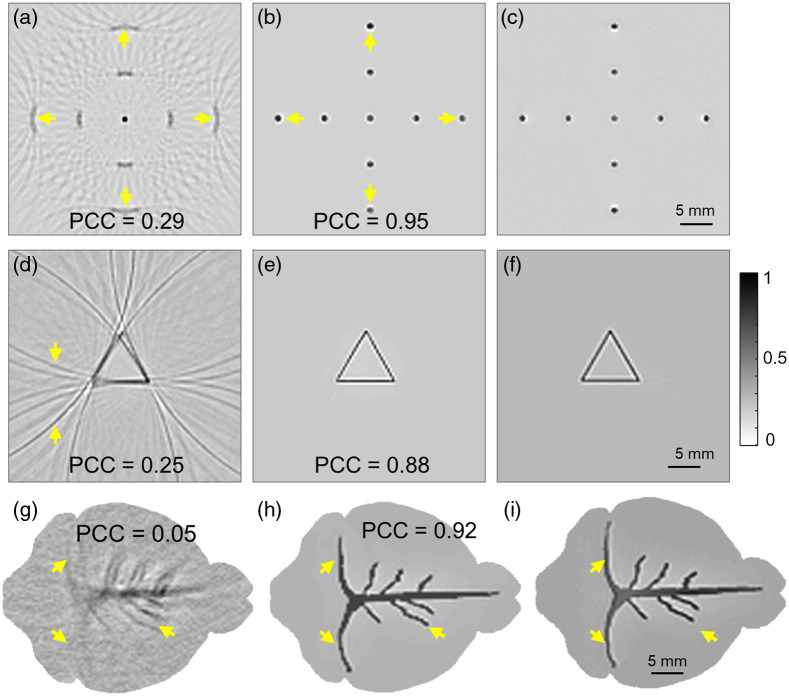
(a)–(c) PAT images of nine-point target numerical phantom (1-UST-PAT configuration): (a) simulated for a scan time of 5 s, (b) reconstructed with HD-UNet, and (c) simulated for a scan time of 8 min (ideal image, or the ground truth). (d)–(f) PAT images of triangular phantom (1-UST-PAT configuration): (d) simulated for a scan time of 5 s, (e) reconstructed with HD-UNet, and (f) simulated for a scan time of 8 min (ideal image, or the ground truth). (g)–(i) PAT images of numerical vessel phantom using (8-UST-PAT configuration): (g) simulated for a scan time of 0.3 s, (h) reconstructed with HD-UNet, and (i) simulated for a scan time of 1.5 s (ideal image, or the ground truth).

The reconstructed PAT images of three numerical phantoms (nine-point target phantom, triangular phantom, and vessel phantom) are shown in Fig. 3. Figure 3(a) shows the PAT image of the nine-point target phantom simulated using 1-UST-PAT configuration for a scan time of 5 s (∼50 A-lines). Figure 3(b) depicts the PAT image of the nine-point target phantom reconstructed using the HD-UNet and Fig. 3(c) shows the ground truth image [1-UST-PAT configuration, 8 min scan time (∼4800 A-lines)]. From Fig. 3(a) it can be noted that point targets are not clearly visible and were also marred by the presence of artifacts arising from the sparse data acquisition at higher scanning speeds. When the HD-UNet has been applied, the artifacts were corrected and the point targets were very well reconstructed [Fig. 3(b)]. Furthermore, the improvement in the tangential resolution over the points can also be noted especially at the farthest point (marked with small yellow arrows) and is very close to the ground truth image [Fig. 3(c)]. As the HD-UNet was not trained on the nine-point targets, the ability of the network to improve the quality nine-point target PAT image signifies its ability on unknown phantom data. Figures 3(d)–3(f) show the PAT images of the triangular phantom obtained using 1-UST-PAT geometry with a scan time of 5 s (∼50 A-lines), with the HD-UNet, and the expected ground truth. The potential of the HD-UNet to preserve the target shape along with improvement in the artifacts (indicated with small yellow arrows) can be observed by comparing Figs. 3(d) and 3(e). The vessel phantom simulated using 8-UST-PAT configuration for a scan time of 0.3 s (30 A-lines per transducer) is shown in Fig. 3(g). Figure 3(h) depicts the PAT image of the vessel phantom reconstructed using the HD-UNet, and Fig. 3(i) shows the ground truth image [8-UST-PAT configuration, 1.5 s scan time (∼1200 A-lines: 150 A-lines per transducer)]. From the comparison of Figs. 3(h) and 3(i), it can be noted that the HD-UNet can produce the image very close to the ground truth even when the scan time was five times less than the ground truth. The improvement noticed over the cerebral venous and veins can be envisaged through visual comparison of the areas indicated by small yellow arrows in Figs. 3(g)–3(i). The higher PCC values of the HD-UNet PAT reconstructed images signify that it plays a very good role by preserving the shape of the target along with improvement in image quality.

### 3.3 Performance of HD-UNet on Experimental Phantom Images

**Fig. 4:**
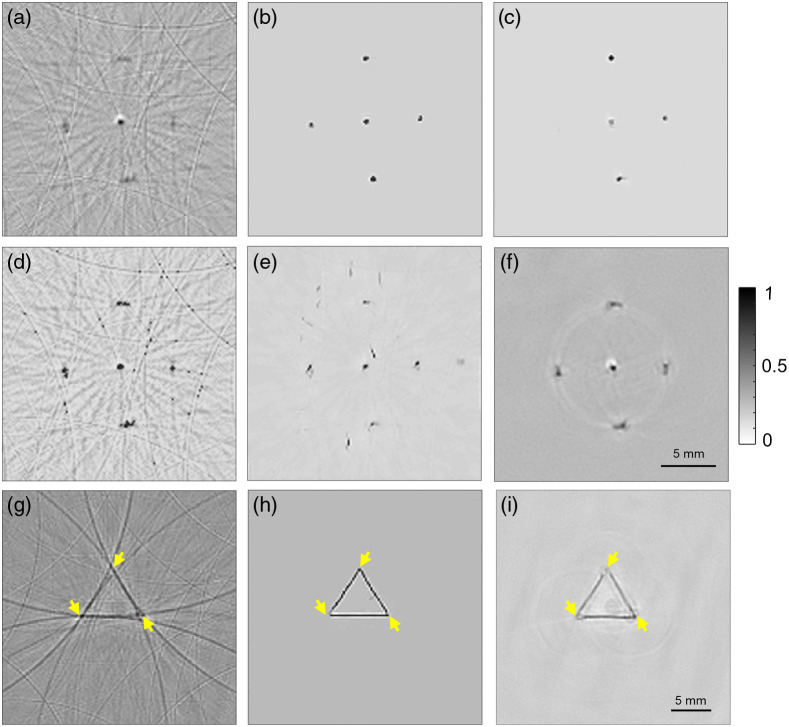
(a)–(f) Reconstructed PAT images of five-point target phantom (1-UST-PAT system): (a) obtained with a scan time of 5 s, (b) reconstructed with HD-UNet, (c) reconstructed with FD-UNet, (d) reconstructed with 2D-DD-UNet, (e) reconstructed with U-Net, and (f) obtained with a scan time of 8 min. (g)–(i) Reconstructed PAT images of triangular horsehair phantom (1-UST-PAT system): (g) obtained with a scan time of 5 s, (h) reconstructed with HD-UNet, and (i) obtained with a scan time of 8 min.

Experimental phantom imaging was performed on the 1-UST-PAT system to evaluate the performance of the HD-UNet at higher imaging speeds. As discussed before, two types of phantoms were utilized for the imaging. Figure 4(a) depicts the five-point target phantom PAT image obtained in a scan time of 5 s. The HD-UNet reconstructed image of the point target phantom is shown in Fig. 4(b). Figures 4(c)–4(e) show the reconstructed PAT image using the FD-Unet, 2D-DD-UNet, and U-Net. Figure 4(f) depicts the PAT image of the point target phantom obtained for a scan time of 8 min. From Fig. 4(f), it can be noted that even though the scan time is high (8 min) the shape of the point targets is not well preserved when its distance from the scanning center increases. When the HD-UNet was employed the shape of the point targets are well preserved along with the removal of artifacts [Fig. 4(b)]. Furthermore, the ability of the HD-UNet to preserve the target shape along with improvement in the quality of the image can be visualized by comparing the PAT image of triangular phantom obtained with a scan time of 5 s [Fig. 4(g)] and the reconstructed image using HD-UNet [Fig. 4(h)]. It can be noted that the edges of the triangular phantom (marked with yellow arrows), which are murkier at higher imaging speeds can be visualized when the HD-UNet was applied, and the resultant image quality is better than that of the PAT image of triangular phantom imaged at a scan time of 8 min [Fig. 4(i)].

**Fig. 5:**
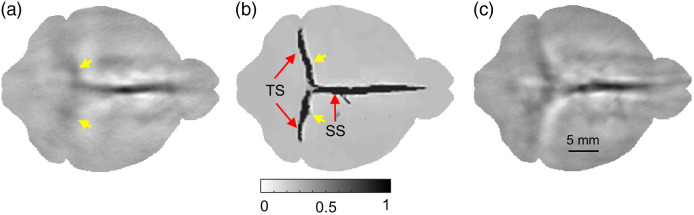
Reconstructed *in vivo* PAT brain images (8-UST-PLD-PAT system): (a) obtained with a scan time of 0.3 s, (b) reconstructed with HD-UNet, and (c) obtained with a scan time of 1.5 s. TS, transverse sinus; SS, sagittal sinus.

The 8-UST-PLD-PAT system described before is used for generating the *in vivo* brain images. Figure 5(a) shows the *in vivo* brain images obtained at a high frame rate of 0.3 s. It can be noted from the images that the cerebral venous sinuses such as the transverse sinuses (TS) are imperceptible (shown with small yellow arrows) due to low SNR at higher scanning speeds, which is a major hindrance for the analysis of various *in vivo* morphological studies such as intracranial hypotension, cerebral hemorrhage, etc. Furthermore, the presence of white artifacts arising from the limited bandwidth detection also limits the visual analysis of sagittal sinus (SS). When the HD-UNet was applied for reconstruction, the cerebral venous sinuses were perceptible (marked with small yellow arrows) along with the removal of artifacts [Fig. 5(b)]. It can also be noted that the HD-UNet also improves the tangential resolution without compromising the image quality in comparison with the conventionally reconstructed *in vivo* brain images obtained using a scan time of 1.5 s [Fig. 5(c)].

## 4 Discussion and Conclusion

In circular view PAT systems, the imaging speed is limited by the artifacts arising from the sparse signal acquisition due to higher scanning speeds. One also needs to factor the laser safety when doing scanning at higher speed with high repetition rate lasers. In the case of using high repetition rate laser with high per pulse energy, we may need to reduce the per pulse energy to be within safety limits. However, most excitation sources with high PRR, such as PLDs, generally have lower per pulse energy (∼3 to 4 mJ per pulse), thus safety concerns are not a major factor. However, the lower per pulse energy results in lower SNR PA signal, thus limiting the imaging speed (even though we can scan at high speed, the poor SNR is a limiting factor). Herein, to improve the frame rate of PAT systems, we proposed a U-Net-based DL architecture called the HD-UNet to reconstruct high-quality PAT images from the data acquired at higher scanning speeds. Simulated datasets were used for optimizing the HD-UNet and its performance was demonstrated on the experimental phantom and *in vivo* images. In the HD-UNet, dense blocks with dilated convolutions are only preferred in the encoding path for aggregating the context without the loss of information, whereas standard convolution dense blocks are used in the decoding path along with residual bridge block to incorporate the artifact removal capability of the FD-UNet with the optimal number of training parameters.^52^ As a standalone network, the FD-UNet and 2D-DD-UNets have their own merits such as artifact removal [Fig. 4(c)] and higher attention to context information [Fig. 4(d)]. These merits were incorporated in a single network (HD-UNet) using dilated dense blocks in the encoding and standard dense blocks in the decoding path of the U-Net [Fig. 4(b)]. The improvements obtained through these extensional techniques on the standard U-Net can be visualized by comparing Figs. 4(b) and 4(e). Furthermore, the application of the proposed HD-UNet can be extended to other types of phantoms if the optimization dataset is curated to the intended application. For phantoms analogous to point sources and triangular shapes, the optimization dataset provided is sufficient and this fact is exemplified by the performance of HD-UNet on nine-point target simulated phantom images [Figs. 3(a)–3(c)]. However, there still exist scenarios where the generation of simulated datasets close to the intended application is unviable. In such cases, training the HD-UNet with a mix of simulated and experimental datasets will help to reap the benefits of both simulated and experimental scenarios. The performance of the HD-UNet can be further enhanced if we use both the optical absorption and acoustic pressure maps for the optimization. Furthermore, instead of using simple delay-and-sum beamformer, one can use multiview Hilbert transform based delay-and-sum approach to obtain unipolar PAT images.^57^^,^^58^ Although it has been widely applied to PAT systems employing array transducers, the application of the multiview Hilbert transform approach on single-element UST-based PAT systems can also be explored. In general, DL is a data-driven approach and the generation of datasets for optimizing the network can be time-consuming. This limitation on the rate of simulated dataset generation can be hastened by using GPUs for simulation instead of CPUs. Another limitation that persists with DL-based approaches is the time taken for training and it increases with the size of the datasets. Thus, it is important to optimize the size of data according to the performance of the model. An alternative approach to reduce the training time is to implement distributing training over multiple GPUs. As the field of GPUs is rapidly evolving, the institution of GPUs with higher Compute Unified Device Architecture (CUDA) cores will also significantly improve the optimization rate of the DL models.

High frame rate (high-speed imaging) PAT imaging with a low-cost setup is challenging. Without using any expensive array transducer and bulky parallel data acquisition hardware/electronics, achieving faster PAT imaging speed is critical for many dynamic imaging applications. At present, the imaging speed of single- and multi-UST-PAT systems is still limited to 30 (s/frame) and 0.5 (s/frame) due to the marring of images by blurring and streaking artifacts (arising from the sparse data acquisition and low SNR) at higher imaging speeds. Thus, to improve the frame rate of PAT systems based on single-element transducers, we developed a U-Net-based DL architecture called the HD-UNet. The HD-UNet comprises dense blocks with dilated convolution in the downsampling layers and standard dense blocks in the up-sampling layers to reconstruct the PAT images from fast-scanning acquired data. For optimizing the HD-UNet, simulated numerical phantoms were used and its performance was evaluated on simulated as well as experimental phantom and *in vivo* images. Our experimental results demonstrate that the proposed HD-UNet can improve the frame rate by approximately sixfold in the single-UST-PAT system^22^ and by approximately twofold in multi-UST-PAT systems.^23^ This is the fastest imaging speed reported so far in the literature in single- and multi-UST-PAT systems. In general, the imaging speed in single-UST-PAT and multi-UST-PAT is not limited to 5 and 0.3 (s/frame), respectively, and it can be further improved to 1 and 0.1 (s/frame), respectively, using the method we described here. But its experimental demonstration is unviable at present due to the low-torque stepper motor used in our experimental setup. If the constraints on the torque of the stepper motor can be subsided, imagining at a frame rate of ∼10  Hz (0.1  s/frame, 10 fps) is imminent using multi-UST-PAT systems. In the future, we will be working toward demonstrating a 10 fps PAT imaging system using single element UST. In addition, the proposed HD-UNet can also be easily adapted to other PAT imaging systems with minimalistic changes in the hyperparameters.

In conclusion, we have demonstrated an imaging frame rate of ∼0.2 and ∼3  Hz on single- and multi-UST-PAT systems. Using the HD-UNet, the imaging frame rate can be further improved to ∼1 (single UST-PAT) and ∼10  Hz (multi-UST-PAT). Our future work will be to modify the stepper motor system and demonstrate this.

## Code, Data, and Materials Availability

The source of HD-UNet is available at GitHub: https://github.com/DeeplearningBILAB/High-frame-rate-3-Hz-circular-photoacoustic-tomography-using-single-element-ultrasound-transducer and the datasets can be downloaded from Open Science Framework (OSF): https://osf.io/9xwru.
